# Identification of differentially expressed immune-related genes in patients with systemic lupus erythematosus and the development of a hub gene-based diagnostic model

**DOI:** 10.1186/s40001-025-02953-1

**Published:** 2025-07-30

**Authors:** Quangang Fang, Weili Kong, Huaping Zhou, Yilin Pang, Haiyun Liu

**Affiliations:** 1 Department of Laboratory, Jiangxi Provincial People’s Hospital, The First Affiliated Hospital of Nanchang Medical College, Nangchang, 330000 China; 2 Zhejiang Provincial Key Laboratory of Medical Genetics, School of Laboratory Medicine and Life Sciences, Wenzhou Medical University, Wenzhou, 325035 Zhejiang China

**Keywords:** Systemic lupus erythematosus, IRF7, MX1, EIF2AK2, Immune cell infiltration, Diagnostic model, Bioinformatics analysis

## Abstract

**Background:**

Systemic lupus erythematosus (SLE) is an incurable autoimmune disease that affects body tissues, but it can be managed with medication. Although therapeutic strategies for SLE have advanced, the underlying molecular mechanisms driving disease pathogenesis remain incompletely understood.

**Methods:**

This study analyzed gene expression data from three GEO microarray datasets to explore immunity-related differentially expressed genes (DEGs) in SLE. Using WGCNA, we identified gene modules and integrated them with immune-related DEGs to find candidate hub genes, which were validated using RT-qPCR. We constructed a PPI network and performed gene enrichment analysis to identify nine hub genes through ROC curve analysis. We confirmed the link between these hub genes and immune cells, conducted GSEA, and predicted drugs, miRNAs, and transcription factors (TFs) targeting these genes. LASSO and ROC analyses validated a model using immunity-related DEGs.

**Results:**

The forty immune-related DEGs were identified from a total of 1590 DEGs, 452 WGCN module genes, and 1791 immune genes. Nine hub genes (*MX1*, *OAS1*, *OASL*, *IRF7*, *RSAD2*, *EIF2AK2*, *ISG15*, *IFIH1*, and *STAT1*) were highlighted using Cytoscape and ROC analysis, with an AUC greater than 0.7. RT-qPCR confirmed significant overexpression of all hub genes except *STAT1* in SLE. ssGSEA and GSEA linked these genes to immune cell infiltration and pathways, including "cell cycle" and "RIG-I-like receptor signaling." A diagnostic model with three immune-related hub genes (*MX1*, *IRF7*, and *EIF2AK2*) demonstrated high accuracy (AUC > 0.8) in distinguishing SLE from healthy controls. Additionally, 9 target drugs, 14 target miRNAs, and 23 TFs were identified for these hub genes.

**Conclusions:**

MX1, IRF7, and EIF2AK2 may serve as candidate biomarkers for SLE and warrant further investigation.

**Supplementary Information:**

The online version contains supplementary material available at 10.1186/s40001-025-02953-1.

## Introduction

Systemic lupus erythematosus (SLE) is a chronic autoimmune disease that affects various systems within the body [1]. It involves immune dysfunction, leading to excessive autoantibodies and the deposition of immune complexes in multiple organs [2]. The activation of the classical complement pathway by these immune complexes subsequently generates inflammatory mediators, thereby facilitating tissue damage [3]. A previous study associated organ damage in SLE patients with factors such as age, disease duration, activity level, poor quality of life, severe fatigue, and azathioprine use, while hydroxychloroquine use and better health scores were protective [4]. Recognizing these links helps in treating SLE. The disease has a gradual onset with diverse symptoms [5], and its exact cause is unknown. Current hypotheses propose that factors such as abnormal immune activation, genetic predisposition, environmental influences, and estrogen levels may contribute to the pathogenesis of SLE [5, 6]. Clinical and immunological biomarkers are anticipated to play a crucial role in enhancing the diagnosis, evaluation, and management of SLE [7]. For instance, research by Živković et al. [8] has demonstrated that serum and urinary levels of monocyte chemoattractant protein-1 (MCP-1) are significantly elevated in patients with SLE compared to healthy individuals. Despite these findings, the clinical application of such biomarkers remains limited. Consequently, there is an essential need for the development and validation of novel immune-related biomarkers for routine clinical use in the management of SLE.

Studies on the pathogenesis of SLE have identified two key groups of mediators: type I interferon (IFN-I) and autoantibodies, such as anti-nuclear antibodies and double-stranded DNA antibodies, both of which are essential for diagnosis [2, 9, 10]. Studies indicate that the overactivation of immune cells is associated with SLE, with T cells playing a particularly significant role [11]. In active SLE, the function of Th1 cells decreases while Th2 and B cells become overactive [12]. Th17 cells produce IL-17A, which stimulates the production of chemokines that recruit inflammatory cells, leading to local inflammation [13–15]. IL-17A also leads to aberrant activation of B cells and dendritic cells (DCs), resulting in enhanced autoantibody production, disrupted intracellular signaling, and elevated cytokine (such as IL-2, 4, 10, TNF-α, IFN-γ, etc.) levels [13, 16, 17]. This dysregulated immune activation is believed to contribute significantly to the development and progression of SLE.

Currently, SLE is incurable, with treatment focused on symptom relief and life extension through the use of medications such as non-steroidal anti-inflammatory drugs, corticosteroids, and immunosuppressants [18]. However, these medications can disrupt the immune system and heighten the risk of infections [19]. Therefore, new treatment targets are needed. This study conducted a comprehensive analysis of gene expression in patients with SLE and healthy controls by integrating data from three GEO datasets. Immune-related markers and hub genes were identified through the construction of protein–protein interaction (PPI) networks. The expression of these hub genes was validated using clinical samples through reverse transcription quantitative polymerase chain reaction (RT-qPCR). A diagnostic model was subsequently developed and externally validated using an independent dataset, GSE81622. Additionally, potential therapeutic drugs and microRNAs (miRNAs) targeting these genes were predicted utilizing the miRNET database, thereby suggesting novel treatment strategies for SLE.

## Materials and methods

### Data collection and processing

**Fig. 1:**
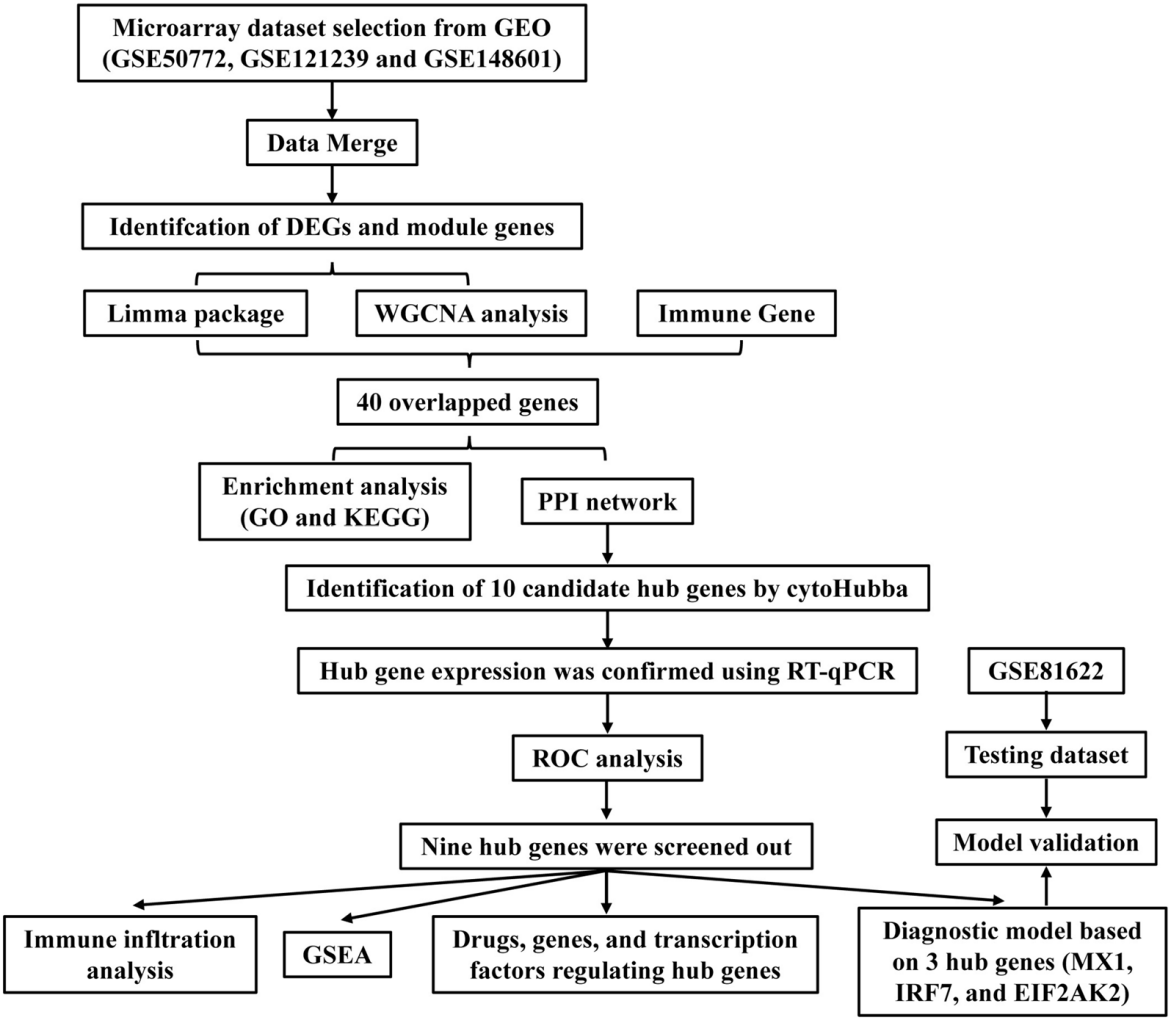
The study flow chart

We analyzed datasets GSE50772, GSE121239, and GSE148601 from the GEO database, which included 375 patients with SLE and 54 healthy controls, along with their clinical data. After merging the gene matrices with a Perl script, we used the R (v4.2.3) package"sva"to reduce batch and platform variability [20], followed by normalization and analysis with "limma". The validation dataset GSE81622 was analyzed using "edgeR". Figure 1 provides a detailed study flow chart. Additionally, we sourced 1791 immune genes from the ImmPort database (https://www.immport.org/shared/home).

### Principal component analysis (PCA)

The repeatability of intra-group data was assessed using Pearson's correlation test for each group. Additionally, sample clustering analysis was employed to evaluate the intra-group data repeatability of the dataset. Statistical analyses were conducted using the R programming language, and the results were visualized utilizing the "ggplot2" package.

### Identification and enrichment of immune-related differentially expressed genes (DEGs) in patients with SLE

The “limma” R package was used to analyze DEGs between SLE patients and healthy controls using the training datasets (GSE50772, GSE121239, and GSE148601). Due to the small log fold change (log FC) values of the microarray data, a DEG filtering threshold of adjusted* p*-value < 0.05 was set. Weighted gene co-expression network analysis (WGCNA) (v1.70-3) was then employed to study the characteristics of SLE patients versus controls [21]. Cluster analysis identified DEG outliers for removal, and Pearson correlation coefficients were calculated to select an optimal soft threshold for a scale-free network. A dynamic tree cutting method created a gene tree map, and the module with the lowest *p*-value was identified as the primary module for further analysis. The identification of immune-related DEGs that influence the immune response in SLE was facilitated by overlaps among DEGs, module genes, and immune-related genes. GO and KEGG enrichment analyses were conducted on these immune-related DEGs using clusterProfiler (v4.2.2) with a significance threshold of *p* < 0.05. Furthermore, OmicCircos (v1.32.0) was used to visualize the genomic distribution and expression levels of immune-related DEGs.

### Identification and correlation analysis of hub genes

The STRING database (https://cn.string-db.org/) was used for protein identification and PPI network construction, while Cytoscape (v3.9.1) was utilized for evaluation and visualization. The MCODE plug-in was employed to identify core network genes. ROC curves were used to assess SLE patients and healthy controls, selecting genes with an area under the curve (AUC) > 0.7. Final hub genes were chosen based on their strong classification performance in training datasets. The correlations between hub gene pairs were analyzed, and scatter plots were created for pairs with the most significant positive and negative correlations.

### The candidate hub genes were confirmed using real‑time quantitative PCR (RT‑qPCR)

Nine SLE patients and seven healthy controls were recruited from Jiangxi Provincial People’s Hospital, all meeting the 1997 ACR SLE criteria [22]. This study included the SLE patients who had an SLEDAI (SLE Disease Activity Index) score greater than 5, while excluding individuals with other autoimmune diseases, serious infections, or cancer. Peripheral blood mononuclear cells (PBMCs) were extracted from human blood treated with EDTA using Ficoll-Paque density gradient centrifugation. Total RNA was extracted with TRIzol, and cDNA was synthesized with HiScript IV RT SuperMix (Vazyme, China). β-Actin, a housekeeping gene, served as an internal reference. RT-qPCR was conducted with ChamQ Universal SYBR qPCR Master Mix (Vazyme, China), and relative expression was calculated using the 2^−△△CT^ method. Primer details are in Supplementary Table S1.

### Immune infiltration analysis and gene set enrichment analysis (GSEA)

Inflammation significantly influences the development of SLE, with immune regulation playing a key role. Using single-sample gene set enrichment analysis (ssGSEA) with GSVA (v1.42.1), we explored the relationship between hub genes and immune cell infiltration. This analysis assessed correlations among 28 immune cell types in the training datasets (GSE50772, GSE121239, and GSE148601). To better understand the roles of these hub genes, samples with both high and low expression levels were analyzed using The Molecular Signatures Database (MSigDB) (http://www.broadinstitute.org/msigdb). The top three pathways were selected for further analysis from the significantly enriched KEGG pathways (adjusted *p*-value < 0.05), based on whether their normalized enrichment scores (NES) were greater than or less than zero.

### Regulatory networks and target drugs of hub genes

We used the miRNET database [23] (https://www.mirnet.ca/) to predict miRNAs and transcription factors (TFs) potentially regulating the identified hub genes. Additionally, the Drug–Gene Interaction database (DGIdb) (https://dgidb.genome.wustl.edu/) [24], which consolidates data on drug–gene interactions from 30 different sources, was utilized to predict drugs that affect the expression of hub genes.

### Construction of a diagnostic model

Using the training dataset (GSE50772, GSE121239, and GSE148601), a diagnostic model was created based on the expression levels of three hub genes: *MX1*, *IRF7*, and *EIF2AK2*. Lasso regression, a type of regression analysis that performs both variable selection and regularization, determined the coefficients for the model. Its validity was assessed by classifying all training samples as either disease or control and verifying this with confusion matrices and ROC curves. The model's performance was further validated using GSE81622 as an external dataset.

### Statistical analysis

Analyses were performed using R (v4.2.3), employing the Wilcoxon test for group comparisons and the Student's *t*-test for RT-qPCR differences. Unless indicated otherwise, a *p*-value of less than 0.05 was considered significant.

## Results

### Identification of DEGs and module genes

**Fig. 2:**
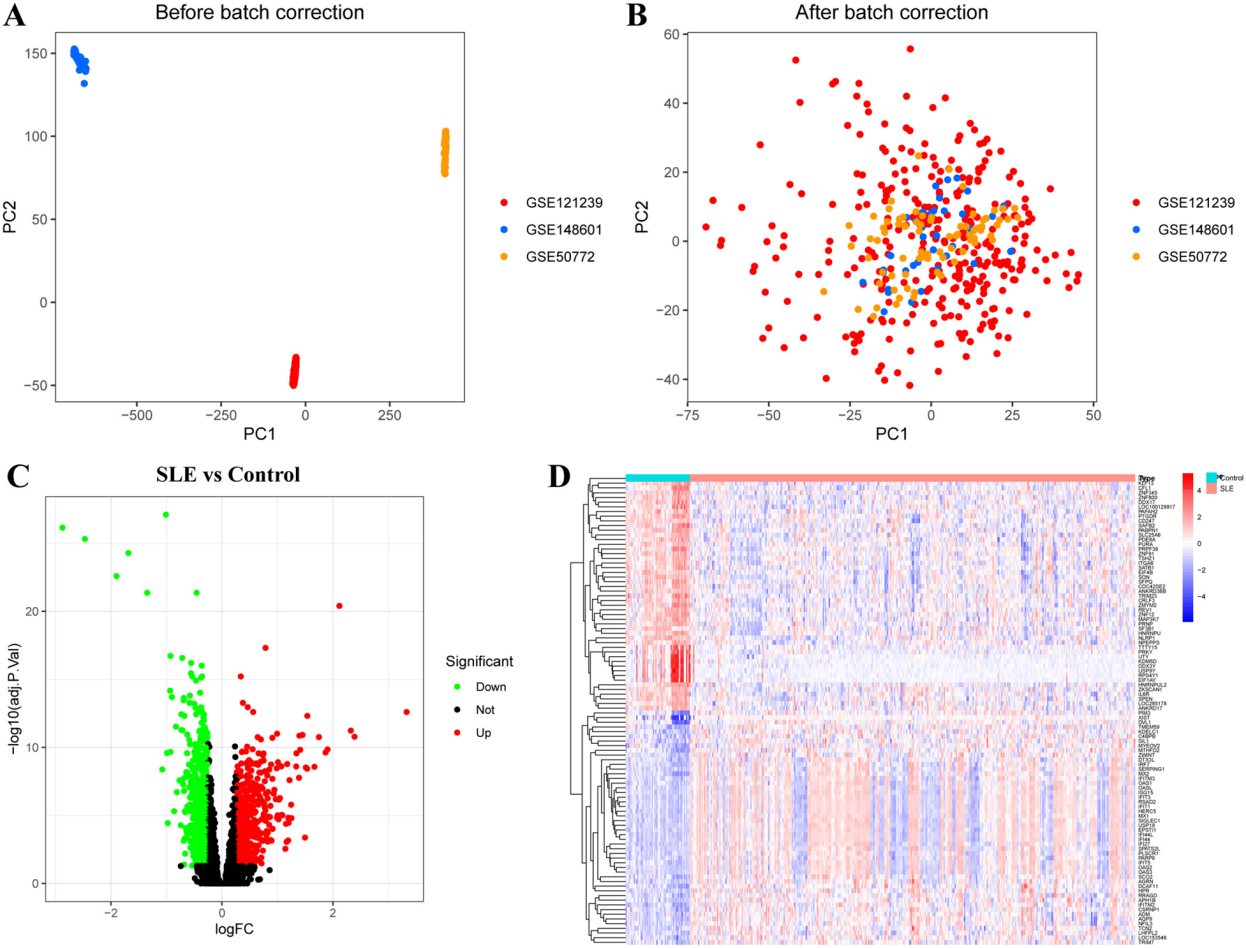
Data preprocessing and identification of differentially expressed genes (DEGs). **A, B** Principal component analyses (PCA) were performed to conduct batch correction on GSE50772, GSE121239, and GSE148601. **A** Before batch correction and **B** after batch correction. **C, D** Volcano plot (**C**) and heatmap (**D**, top 50) of DEGs in peripheral blood mononuclear cells (PBMCs) from patients with SLE and healthy controls. Statistical analyses were conducted using R software (v4.2.3)

**Fig. 3:**
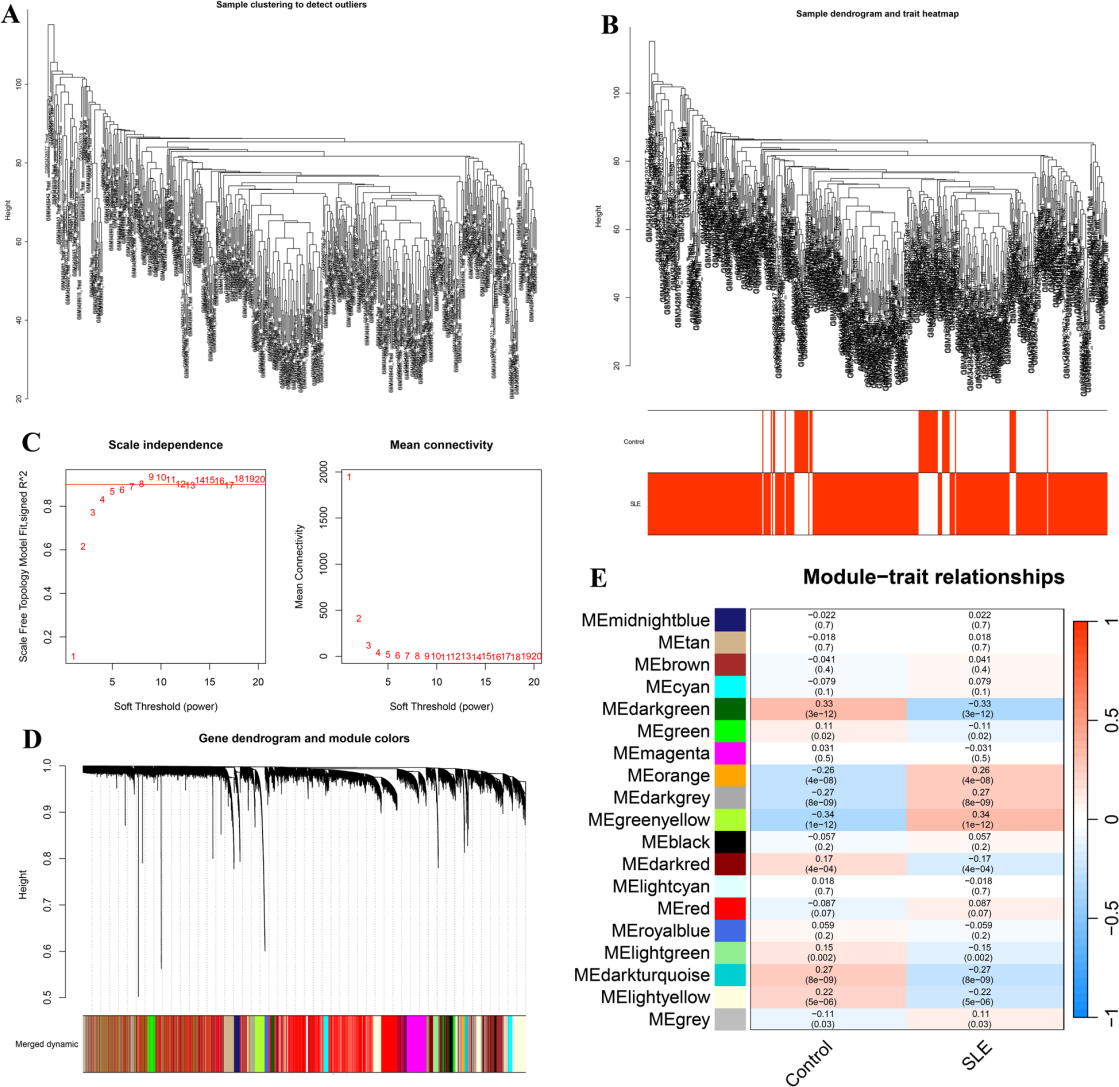
Identification of module genes from DEGs. **A** Outlier sample detection. The gene sets from all samples are contained in the dendrogram. **B** Sample dendrogram and trait heatmap. **C** Soft threshold screening. **D** WGCNA modules. **E** Module-disease correlations. Statistical analyses were conducted using R software (v4.2.3)

Three microarray raw datasets (GSE50772, GSE121239, and GSE148601), including 375 SLE patients and 54 healthy controls, were selected for the identification of DEGs in SLE. The data before (A) and after (B) batch correction are presented in Fig. 1, indicating that the batch effect of the merged data was successfully removed. A total of 1,590 DEGs were screened in PBMCs from SLE patients compared to healthy controls, with 777 genes showing increased expression and 813 showing decreased expression (Fig. 2C). The top 50 most significant genes were used to create a heatmap (Fig. 2D). After removing outlier genes, a weighted gene co-expression cluster analysis included all sample genomes in the tree map (Fig. 3A, B). A scale-free network was constructed using a soft threshold of 8 (*R*^2^ = 0.90) (Fig. 3C), identifying 19 modules (Fig. 3D). The green-yellow module, containing 452 genes, was selected for further analysis based on the lowest *p*-value (Fig. 3E).

### Screening and functional enrichment of immune-related DEGs

**Fig. 4:**
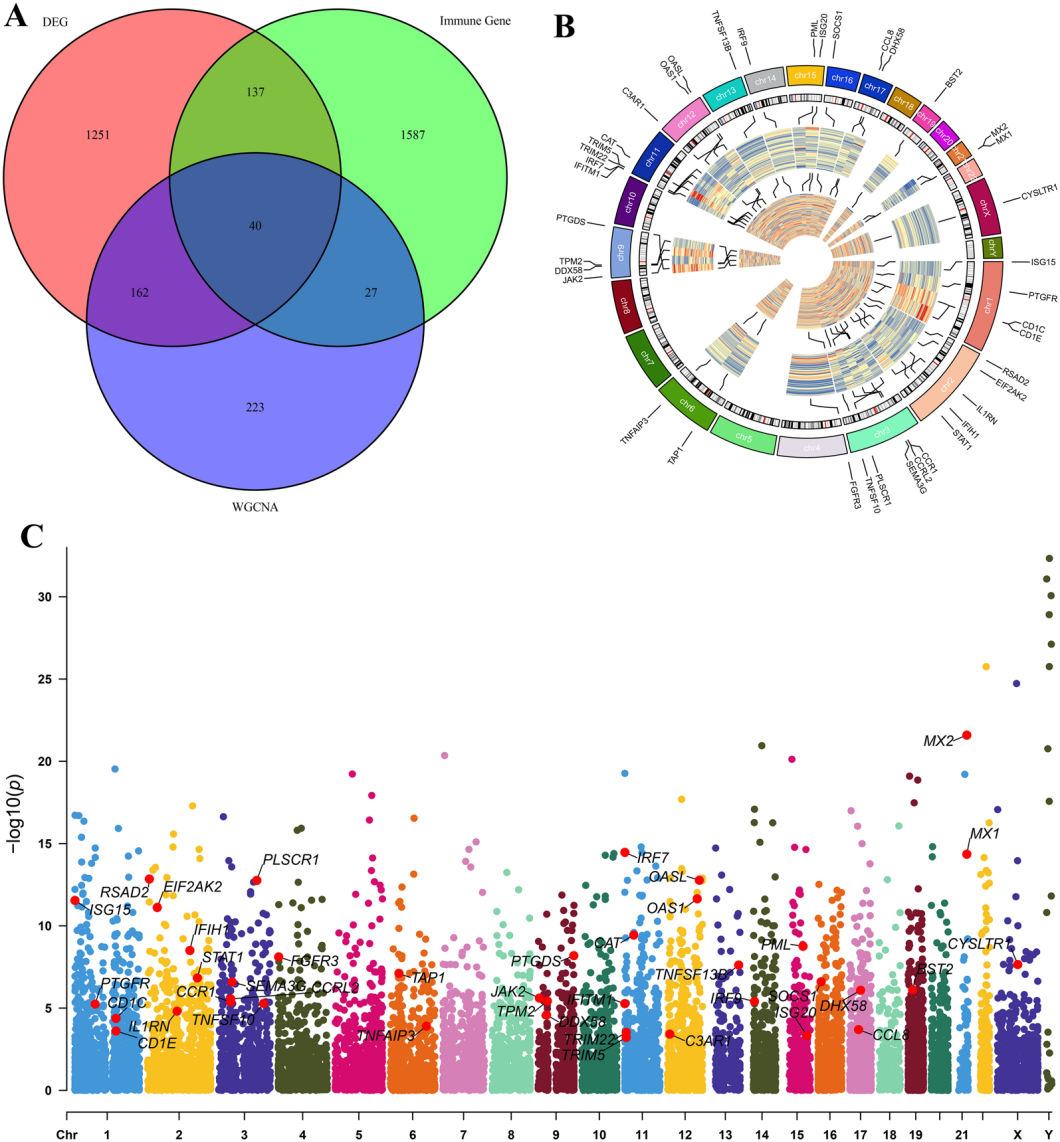
Screening of immune-related DEGs. **A** Venn diagram of immune-related DEGs screening. **B** The expression patterns of these genes in the three merged GEO datasets (GSE50772, GSE121239, and GSE148601) are represented in the inner circular heatmaps. Dark red indicates gene upregulation, while dark blue indicates downregulation. The outer circle of the heatmap corresponds to the control group, and the inner circle corresponds to the disease group. The chromosomes are represented by the outer circle, and lines from each gene highlight their specific locations on the chromosomes. **C** Manhattan plot of immune-related DEGs

An overlap analysis among 1590 DEGs, 1791 immune genes, and 452 module genes identified 40 immune-related DEGs linked to SLE patients (Fig. 4A). The precise cause of SLE is still unclear, but some scientists propose a genetic connection, prompting further research into specific chromosomal sites. Figure 4B, C illustrates the gene expression patterns and chromosomal locations of these DEGs in SLE patients and healthy controls. The results reveal their presence on all chromosomes except for 5, 7, 8, 10, 18, 20, 22, and Y chromosomes, with the highest number of genes located on chromosomes 2, 3, and 11.

**Fig. 5:**
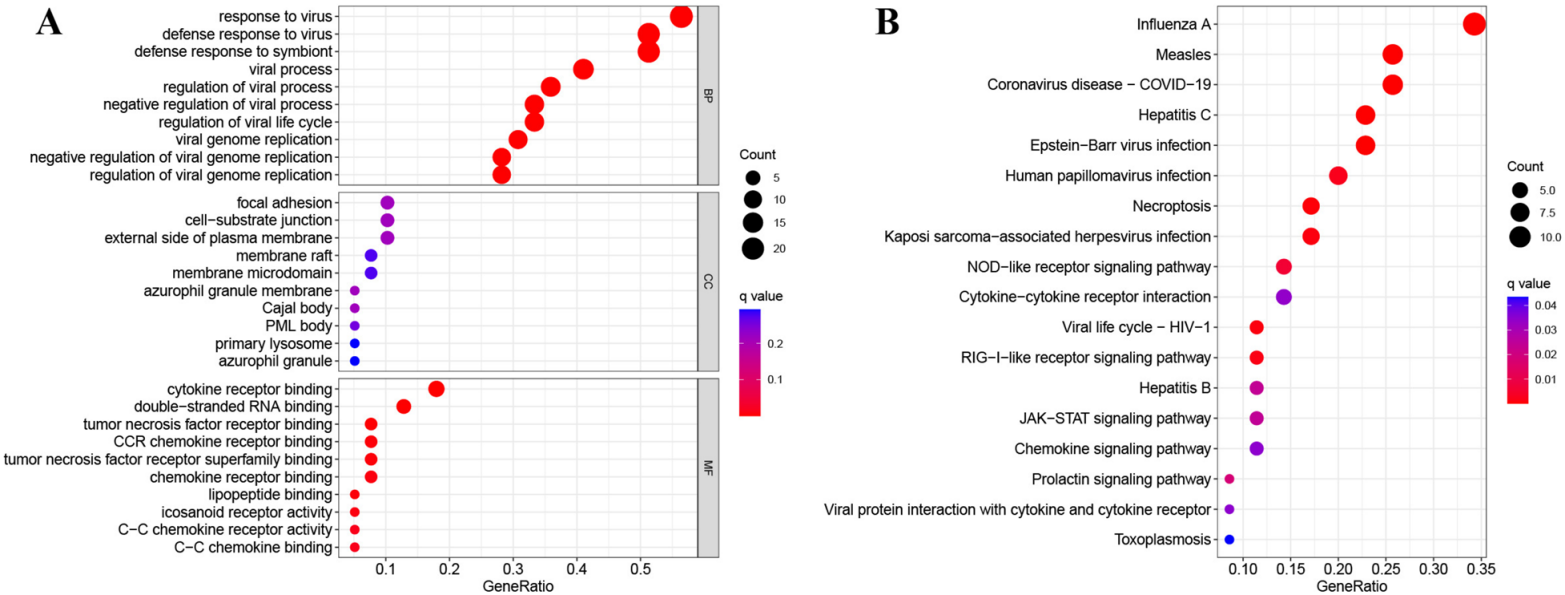
GO (**A**) and KEGG pathway (**B**) enrichment analysis of immune-related DEGs. Statistical analyses were conducted using R software (v4.2.3)

The immune-related DEGs underwent enrichment analysis, and the GO analysis covered cellular component (CC), molecular function (MF), and biological process (BP). The BP included responses to viruses and symbionts, the CC encompassed focal adhesion and cell-substrate junctions, and the MF involved cytokine receptor and double-stranded RNA binding (Fig. 5A). The KEGG pathway analysis highlighted pathways such as influenza A, measles, COVID-19, NOD-like receptor signaling, cytokine-cytokine receptor interaction, and RIG-I-like receptor signaling (Fig. 5B).

### The hub gene was identified through co-expression analysis and validated by RT-qPCR

**Fig. 6:**
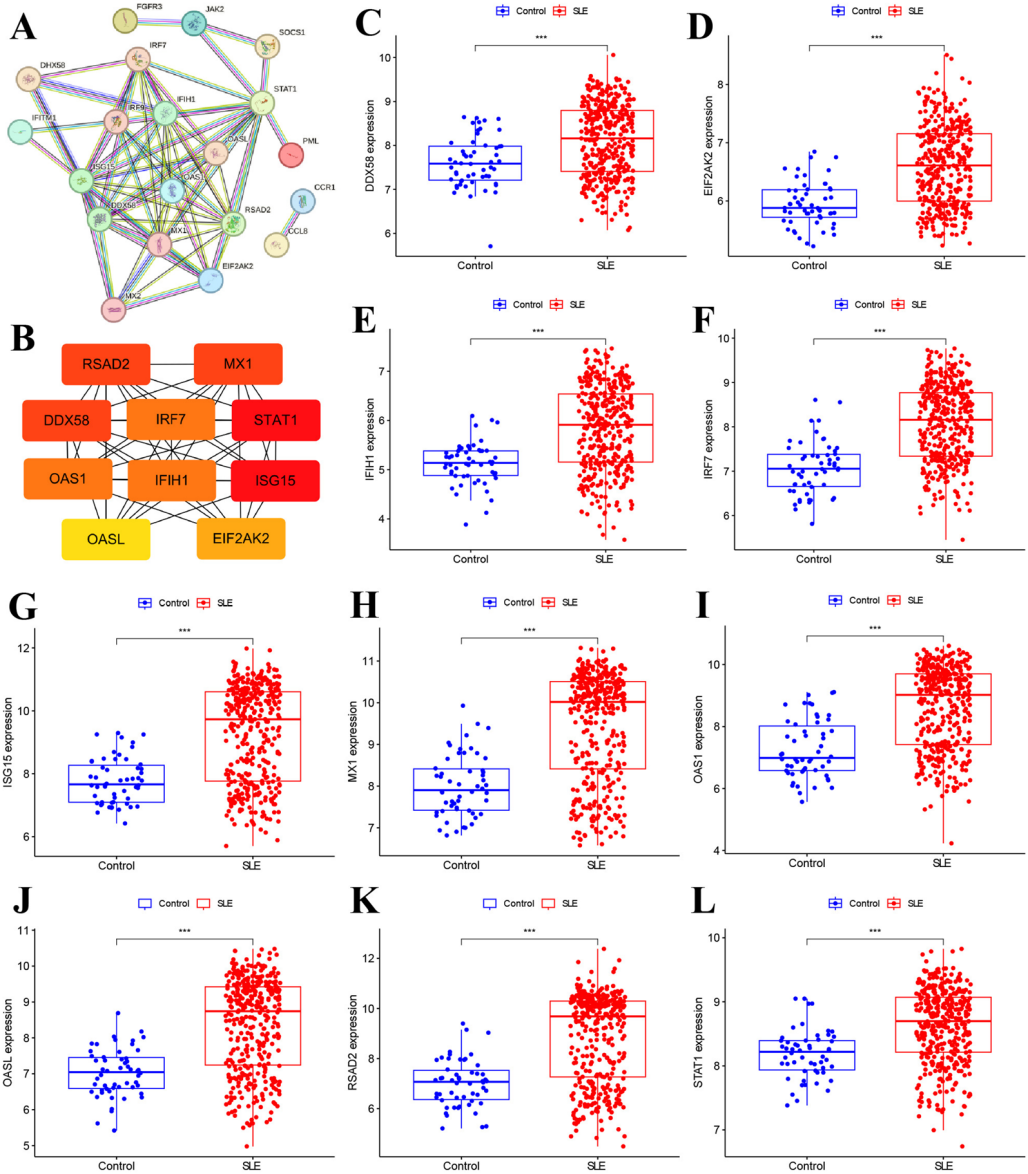
Identifying candidate hub genes using co-expression analysis. **A** A protein–protein interaction (PPI) network was built from immune-related DEGs. **B** Hub subnetworks were formed through Cytoscape. **C-L** The expression levels of candidate hub genes in PBMCs from SLE patients (*n* = 375) and healthy controls (*n* = 54) were analyzed in the training dataset. Data are presented as median ± standard error (SE). Statistical comparisons between the two groups were conducted using the Wilcoxon test. SLE: systemic lupus erythematosus. ***, *p* < 0.001

**Fig. 7:**
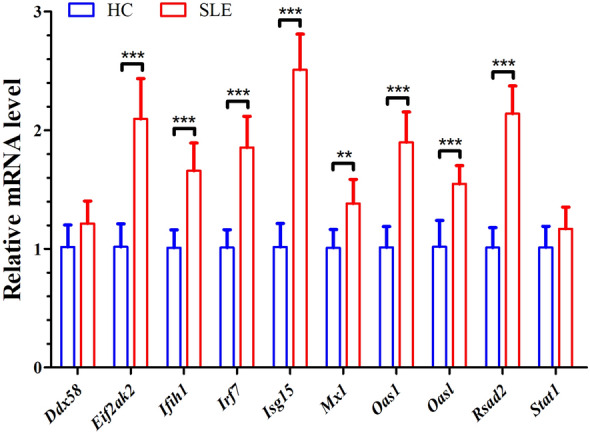
The expression levels of 10 candidate hub genes in PBMCs from patients with SLE (*n* = 9) were validated using RT-qPCR, compared to healthy controls (*n* = 7). Data are presented as the mean ± standard deviation (SD). Statistical comparisons between the two groups were conducted using the Student's *t*-test. HC: healthy controls; SLE: systemic lupus erythematosus. **, *p* < 0.01; ***, *p* < 0.001

**Fig. 8:**
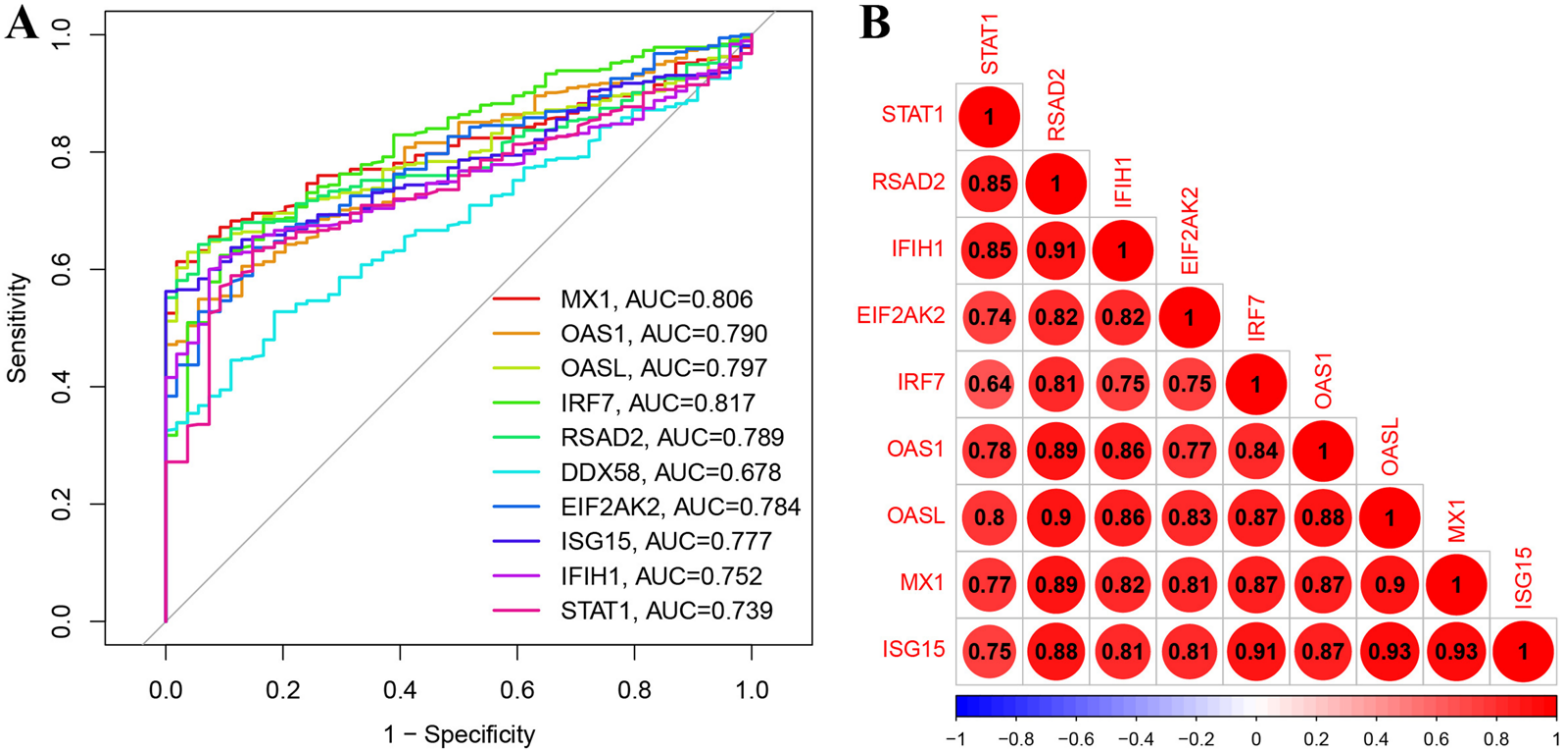
Examine the performance of the ten candidate hub genes and investigate the co-expression relationships among them. **A** ROC curve analysis was used to assess the diagnostic performance of ten candidate hub genes. The top nine genes, based on their AUC (area under the curve) scores for distinguishing SLE (*n* = 375) from healthy controls (*n* = 54), were selected for further investigation. **B** The relationships between the expression levels of various hub gene pairs were analyzed using the Spearman correlation method

We constructed the PPI network utilizing the STRING network online analysis tool and employed the Cytoscape software for network visualization. A sub-network comprising 10 hub genes—*RSAD2*, *MX1*, *DDX58*, *IRF7*, *STAT1*, *OAS1*, *IFIH1*, *ISG15*, *OASL*, and *EIF2AK2*—was identified (Fig. 6A, B). Analysis of the network revealed that *STAT1* and *ISG15* exhibited the highest connectivity with other genes, followed by *RSAD2*, *MX1*, and *DDX58* (Fig. 6B). Subsequently, we assessed the expression levels of these 10 hub genes in PBMCs, finding significant upregulation in the SLE group compared to healthy controls within the training dataset (Fig. 6C–L). In this study, peripheral blood samples were collected from patients with SLE (*n* = 9) and healthy individuals (*n* = 7) to isolate PBMCs and extract RNA. The subsequent RT-qPCR analysis revealed that, apart from *DDX58* and *STAT1*, the expression levels of the other eight hub genes were notably different in SLE patients compared to healthy controls. Notably, all these genes exhibited significantly elevated expression levels, suggesting a strong association with the pathogenesis and progression of SLE (Fig. 7 and Supplementary Tables S2–3). ROC curve analysis was performed to determine the diagnostic capability of each hub gene for SLE. Among the nine hub genes, *MX1*, *OAS1*, *OASL*, *IRF7*, *RSAD2*, *EIF2AK2*, *ISG15*, *IFIH1*, and *STAT1*, those with an AUC greater than 0.7 were selected for further analysis (Fig. 8A). Pairwise co-expression analysis of these nine hub genes revealed positive correlations, with *ISG15* showing the strongest positive correlation with *MX1* and *OASL* (correlation coefficient = 0.93), followed by the correlation between *MX1* and *OASL* (correlation coefficient = 0.9) (Fig. 8B and Supplementary Figs. S1–S3).

### Correlation between immune cell infiltration scores and hub gene expression levels

**Fig. 9:**
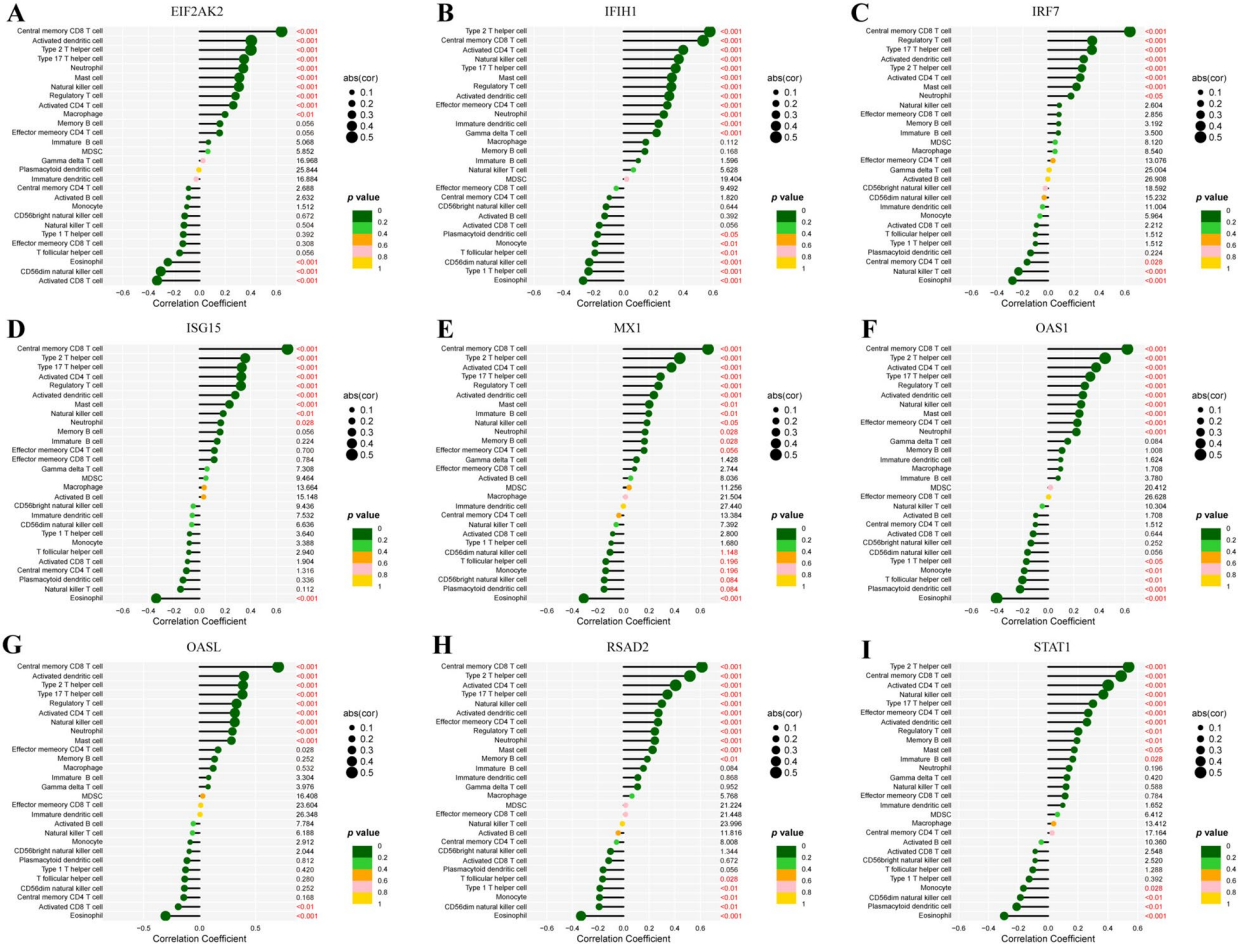
The association between immune cell infiltration scores and hub gene expression levels was analyzed using the Spearman correlation method. **A** *EIF2AK2*, **B** *IFIH1*, **C** *IRF7*, **D** *ISG15*, **E** *MX1*, **F** *OAS1*, **G** *OASL*, **H** *RSAD2*, **I** *STAT1*

To investigate the role of hub genes in immune regulation, we analyzed their association with 28 immune cell infiltrations. The expression of hub genes was positively correlated with central memory CD8^+^ T cells, activated dendritic cells, type 2 and type 17 T helper cells, neutrophils, mast cells, regulatory T cells, and activated CD4^+^ T cells. A significant positive relationship was observed with central memory CD8^+^ T cells, type 2 and type 17 T helper cells, activated dendritic cells, regulatory T cells, activated CD4^+^ T cells, and mast cells, whereas eosinophils showed a significant negative relationship. No significant correlation was found with MDSCs (Fig. 9).

### Gene set enrichment analysis (GSEA)

**Fig. 10:**
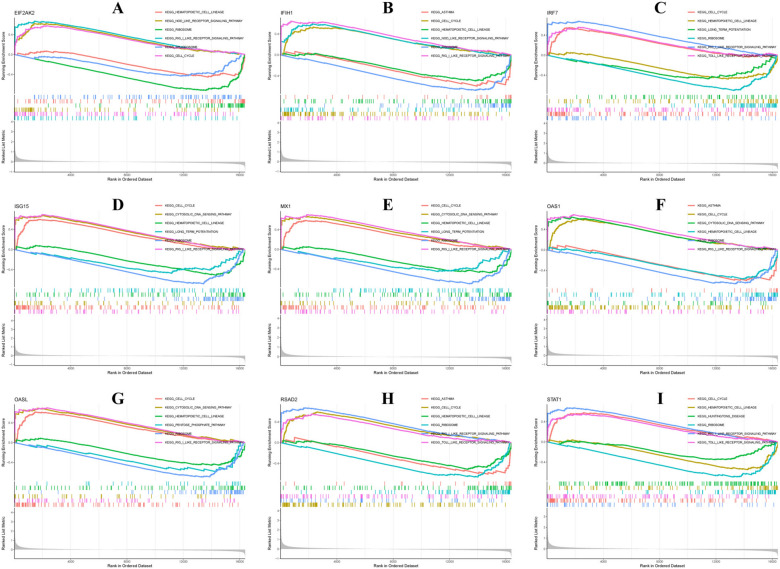
Gene set enrichment analysis for **A** *EIF2AK2*, **B** *IFIH1*, **C** *IRF7*, **D** *ISG15*, **E** *MX1*, **F** *OAS1*, **G** *OASL*, **H** *RSAD2*, and **I** *STAT1*. A gene set was deemed to be enriched when the normalized *p*-value was less than 0.05 and the false discovery rate (FDR) score was also below 0.05

The molecular function of the hub genes was analyzed using GSEA, revealing their overexpression and upregulation in the "Cell cycle" and "RIG-I-like receptor signaling pathway", but downregulation in the "Ribosome" and "Hematopoietic cell lineage" pathways. Specifically, *RSAD2*, *STAT1*, and *IRF7* were upregulated in the "Toll-like receptor signaling pathway", while *OASL* and *ISG15* were upregulated in the "Cytosolic DNA sensing pathway" (Fig. 10).

### Drugs, genes, and transcription factors regulating hub genes

**Fig. 11:**
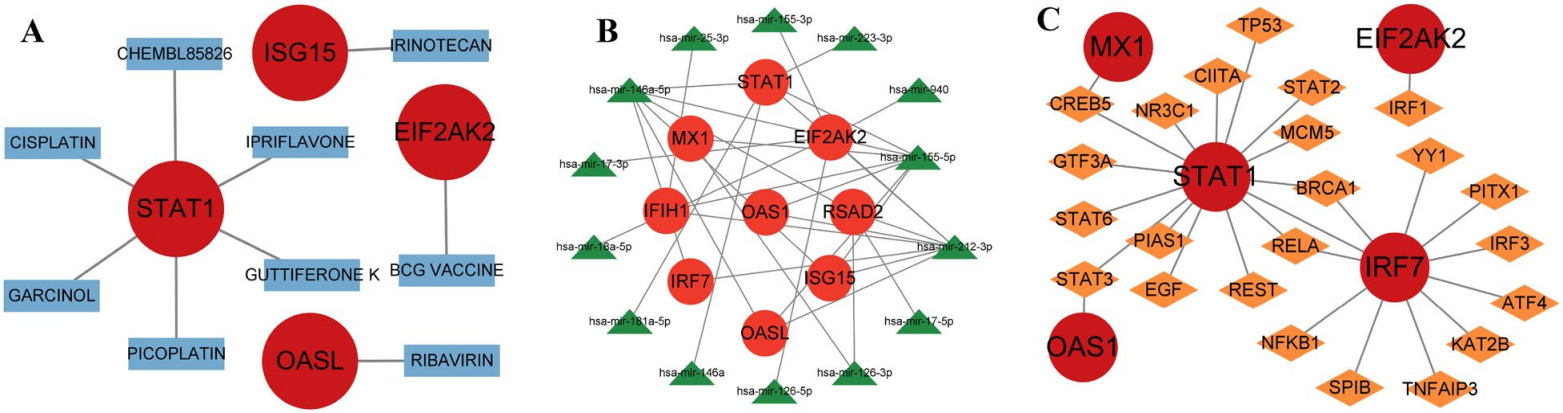
Drugs, genes, and transcription factors that influence hub genes. **A** Small-molecule drugs that affect the expression or function of hub genes. **B** MicroRNAs that target hub genes. **C** Transcription factors that regulate the expression of hub genes. The circles denote hub genes, while the rectangles signify small-molecule drugs. The triangles represent microRNAs, and the rhombuses symbolize transcription factors

The drug–gene interaction database helped us discover nine small-molecule drugs that have the potential to influence hub genes. *ISG15* is linked to IRINOTECAN; *EIF2AK2* is linked to the BCG vaccine; *OASL* is linked to Ribavirin; and *STAT1* is linked to six drugs (Chembl85826, Ipriflavone, Guttiferone k, Picoplatin, Garcinol, and Cisplatin). We then used the miRNet database to create a hub genes-miRNA and hub genes-TF network, identifying 14 target miRNAs and 23 TFs. For instance, BRCA1 and RELA regulate *STAT1* and *IRF7*, CREB5 regulates *MX1* and *STAT1*, and STAT3 regulates *STAT1* and *OAS1*. Among the hub genes, *STAT1* had the most predictive transcription factors (Fig. 11).

### Diagnostic model based on 3 hub genes

**Fig. 12:**
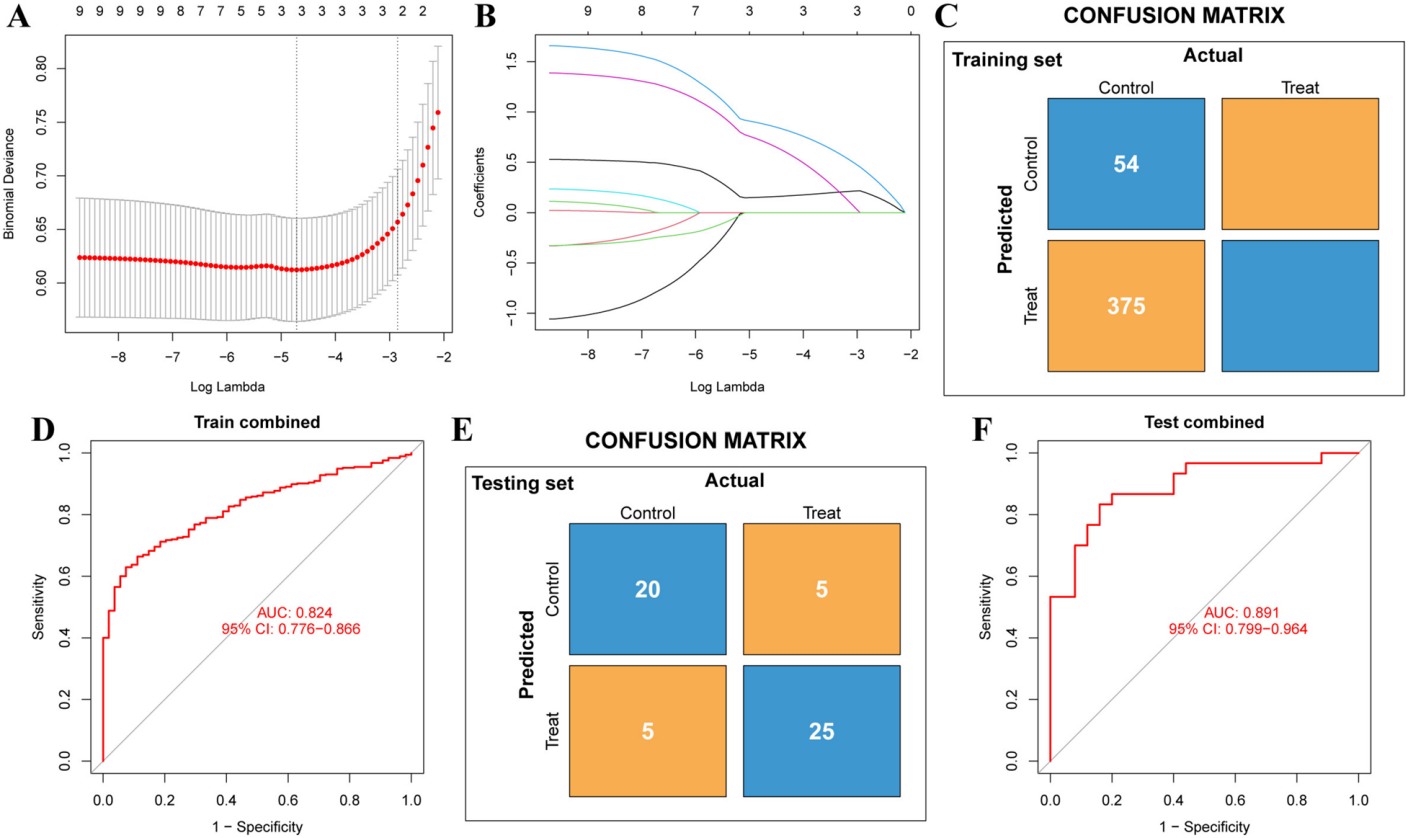
Diagnostic model based on 3 hub genes. **A** The LASSO regression analysis involved ten-fold cross-validation, with error bars representing the standard error (SE). Dotted vertical lines denote the optimal lambda value, which indicates the best level of regularization for the model. **B** Profiles of 3 hub genes' LASSO coefficients. **C** The confusion matrix for the training set includes data from 375 SLE patients and 54 healthy controls. **D** ROC curve for training set. **E** Confusion matrix for the validation set (GSE81622 dataset), which includes 30 SLE patients and 25 healthy controls. **F** ROC curve for validation set. Statistical analyses were conducted using R software (v4.2.3)

Based on the expression profiles of selected hub genes, we developed and validated a diagnostic model to distinguish SLE patients from healthy controls (Fig. 12). The optimal model from the training data had a lambda.min of 0.008973, with gene coefficients of *MX1*, *IRF7*, and *EIF2AK2* being 0.154605, 0.877366, and 0.701475, respectively (Fig. 12A, B). It showed a specificity of 0.93, a sensitivity of 0.63, and an AUC of 0.824 (Fig. 12C, D), indicating good discrimination. Validation with the GEO dataset GSE81622 confirmed the model's effectiveness, showing a specificity of 0.84, a sensitivity of 0.83, and an AUC of 0.891 (Fig. 12E, F).

## Discussion

SLE is a common autoimmune disorder characterized by the production of autoantibodies and damage to organs [25]. While its exact cause remains unclear, it is thought to involve the overactivation of T and B cells and the deposition of antigen-antibody complexes [26]. Advances in high-throughput sequencing technologies, such as transcriptome, methylation, and single-cell sequencing, have enhanced our understanding of autoimmune diseases [27–29]. In the big data era, analyzing shared sequencing data allows researchers to quickly identify disease patterns and hub genes, streamline research directions, minimize errors, and accelerate the discovery of disease mechanisms and targeted therapies. Given the limited big data analysis on SLE, we employed bioinformatics to examine SLE patient samples, identify biomarkers, and explore potential disease mechanisms.

SLE, an autoimmune disease, influences various organs and systems. Prior bioinformatics studies have separately evaluated transcriptomic data from PBMCs and renal tubulointerstitial tissues in patients with SLE [30, 31]. In contrast, our study uniquely combines expression profiles from PBMCs of patients with varying SLE severity. This approach better identifies key SLE molecules and provides a convenient method for early diagnosis and treatment through the screening of hub genes.

While several hub genes (e.g., *IRF7*, *MX1*, *STAT1*) have been previously identified as potential diagnostic biomarkers in SLE studies [32–34], this research presents several novel contributions: 1) the integration and analysis of three GEO microarray datasets were conducted to identify differentially expressed immune-related genes in patients with SLE and to develop a diagnostic model based on hub genes; 2) external validation of findings using the independent GSE81622 dataset; and 3) development and evaluation of a diagnostic model employing LASSO regression and ROC analysis to assess performance. In this study, screening of hub genes and GO analysis showed that immune-related DEGs are involved in immune pathways, such as virus response. KEGG analysis confirmed that immune-related DEGs play roles in viral and inflammatory responses, indicating their connection to immune function. We pinpointed nine hub genes that show higher expression levels in the PBMCs of SLE patients compared to healthy controls. Except for *STAT1*, the other eight genes showed significant overexpression in SLE, as confirmed by RT-qPCR. These genes are associated with increased disease activity and correlate with the infiltration of various immune cells. This suggests that their high expression may exacerbate SLE by intensifying the immune response. Notably, *MX1*, *IRF7*, and *EIF2AK2* were selected via LASSO regression to create a diagnostic model that effectively distinguishes SLE patients from healthy individuals, a finding validated in a test dataset (GSE81622). This indicates that PBMC expression levels of these genes could facilitate quicker and more accurate SLE diagnosis, though further validation with extensive clinical samples is needed.

Interferon-induced, double-stranded RNA-activated protein kinase (EIF2AK2), activated by viral infections, phosphorylates the eIF2 alpha subunit to inhibit translation and viral replication [35] and plays a key role in inflammasome activation [36]. Wei et al. [37] showed berberine targets EIF2AK2 for its anti-inflammatory effects, suggesting that inhibiting EIF2AK2 dimerization could treat inflammation-related disorders. Suzuki et al. [38] found higher *EIF2AK2* expression in SLE patients than in healthy or rheumatoid arthritis (RA) patients, affecting T cell responses. Ge et al. [39] reported that EIF2AK2 influences immune and SLE-related gene transcription. Liu et al. [40] discovered that circRNAs, which inhibit EIF2AK2, are reduced in SLE patients' PBMCs, and their overexpression can reduce EIF2AK2 activation, linking circRNAs to SLE. Future research should focus on collecting clinical samples and conducting *in vivo* experiments to assess EIF2AK2 as a diagnostic and therapeutic target for SLE.

We observed high AUC values (up to 0.8) for *MX1* and *IRF7* in the diagnostic model, which prompted us to investigate their roles in the pathogenesis of SLE. IRF7 plays a vital role in regulating type I interferon (I-IFN) immune responses, which are key to protecting against DNA and RNA viruses [41, 42]. It manages the transcription of type I-IFN and IFN-stimulated genes by binding to specific promoter elements [43, 44]. A genetic variant (SNP rs1131665) in *IRF7* has been linked to SLE, possibly affecting IRF7's function and activating the I-IFN pathway, thereby contributing to the disease [45, 46]. Pan et al. [47] identified that SLE patients exhibited significantly higher *IRF7* expression compared to healthy controls in five GEO datasets (GSE121239, GSE100163, GSE61635, GSE45291, and GSE110169). Kim et al. [48] showed that IRF7 plays a role in the virus-mediated mechanisms of SLE and is linked to colony-forming unit-monocyte and granulocyte levels. Wang et al. [49] proposed that the effectiveness of Langchuangding (LCD) therapy in treating SLE may be due to its impact on the TLR7-IRF7-IFNα pathway, suggesting that IRF7 could be a potential target for SLE treatment. Therefore, exploring IRF7 could provide new insights into the pathogenesis of SLE.

The Mx (Myxovirus resistant) gene, known for its anti-myxovirus properties, was discovered in 1963 [50]. Horisberger et al. [51, 52] identified a 75 kD polypeptide, termed Mx protein, produced only after IFN-α/β induction [53]. This protein, a GTPase enzyme, is part of a dynamin superfamily and plays a crucial role in antiviral activity [54]. The human *Mx1* (*MxA*) gene, also known as *IF178*, encodes a member of the dynein family that possesses GTPase activity and is resistant to several RNA viruses, activated by interferon via the JAK/STAT pathway [55]. While numerous studies have reported the upregulation of *MX1* in SLE [56–58], the precise underlying mechanisms remain largely unexplored, highlighting the need for further research in this area.

In this study, the diagnostic model constructed using *MX1*, *IRF7*, and *EIF2AK2* demonstrated AUC values that surpassed those of individual genes, such as *MX1*, *IRF7*, or *EIF2AK2*, regardless of whether the training set or the testing set was employed. In conclusion, both *MX1* and *IRF7*, as well as the diagnostic model, exhibit potential as diagnostic markers for SLE. Nonetheless, further investigation is warranted to elucidate the relationship between the elevated expression of MX1 and IRF7 and the onset and progression of SLE, utilizing cellular function experiments and animal models.

Our study acknowledges several limitations. Despite employing advanced bioinformatics techniques and validating our results using RT-qPCR in clinical samples, the roles of hub genes, immune cell infiltration, and signal transduction in SLE remain speculative. To corroborate these findings, further functional validation experiments are essential. These could involve the application of small-molecule inhibitors targeting hub genes, as well as gene knockdown and overexpression studies conducted *in vitro* or in animal models. Additionally, it is important to note that this study utilized microarray data rather than RNA-seq data, which may introduce potential batch effects due to the merging of three datasets. The number of clinical samples used for RT-qPCR validation was limited, and there was a lack of age and sex matching in the validation cohorts. Addressing these limitations in future studies could lead to the development of novel strategies for the diagnosis and treatment of SLE.

## Conclusion

In conclusion, this study identified ten immune-related hub genes in SLE patients via bioinformatics, validated by RT-qPCR. Nine genes showed an AUC above 0.7 in ROC analysis. A diagnostic model using three genes (*MX1*, *IRF7*, *EIF2AK2*) was developed through LASSO regression and ROC analysis, effectively distinguishing SLE patients from healthy individuals in the GEO dataset GSE81622. However, further experimental validation is necessary to confirm the RT-qPCR results in a larger, demographically matched cohort. Overall, this research provides a novel theoretical foundation for elucidating the pathogenesis of SLE and offers potential diagnostic markers and models. Future *in vitro* and *in vivo* studies are needed to explore the unreported links between some of the nine target drugs and SLE, which will clarify the role of three hub genes in SLE progression and suggest new treatment targets.

## Supplementary Information

Additional file 1: Fig. S1. Correlation analysis. (A) Correlation between IFIH1 and EIF2AK2 expression. (B) Correlation between EIF2AK2 and ISG15 expression. (C) Correlation between EIF2AK2 and STAT1 expression. (D) Correlation between IFIH1 and STAT1 expression. (E) Correlation between IRF7 and EIF2AK2 expression. (F) Correlation between IRF7 and IFIH1 expression. (G) Correlation between IRF7 and ISG15 expression. (H) Correlation between RSAD2 and IRF7 expression. (I) Correlation between IRF7 and STAT1 expression

Additional file 2: Fig. S2. Correlation analysis. (A) Correlation between ISG15 and IFIH1 expression. (B) Correlation between ISG15 and STAT1 expression. (C) Correlation between MX1 and EIF2AK2 expression. (D) Correlation between MX1 and IFIH1 expression. (E) Correlation between MX1 and IRF7 expression. (F) Correlation between MX1 and ISG15 expression. (G) Correlation between MX1 and OAS1 expression. (H) Correlation between MX1 and OASL expression. (I) Correlation between MX1 and RSAD2 expression. (J) Correlation between MX1 and STAT1 expression

Additional file 3: Fig. S3. Correlation analysis. (A) Correlation between OAS1 and EIF2AK2 expression. (B) Correlation between OAS1 and IFIH1 expression. (C) Correlation between OAS1 and IRF7 expression. (D) Correlation between OAS1 and ISG15 expression. (E) Correlation between OAS1 and OASL expression. (F) Correlation between OAS1 and RSAD2 expression. (G) Correlation between OAS1 and STAT1 expression. (H) Correlation between OASL and EIF2AK2 expression. (I) Correlation between OASL and IFIH1 expression. (J) Correlation between OASL and IRF7 expression. (K) Correlation between OASL and ISG15 expression. (L) Correlation between OASL and RSAD2 expression. (M) Correlation between OASL and STAT1 expression. (N) Correlation between RSAD2 and EIF2AK2 expression. (O) Correlation between RSAD2 and IFIH1 expression. (P) Correlation between RSAD2 and ISG15 expression. (Q) Correlation between RSAD2 and STAT1 expression

Additional file 4: Table S1. Sequences of Primers for the RT-qPCR

## Data Availability

The datasets (GSE50772, GSE121239, GSE148601, and GSE81622) used in this study are accessible in the GEO repository (https://www.ncbi.nlm.nih.gov/geo/).
