# Supplementary material for: Identification of differentially expressed immune-related genes in patients with systemic lupus erythematosus and the development of a hub gene-based diagnostic model
Source: Eur J Med Res. 2025 Jul 30;30:689. doi: 10.1186/s40001-025-02953-1 (PMC12308984; doi:10.1186/s40001-025-02953-1)
Supplement: Supplementary file 1 — Additional file 1: Fig. S1. Correlation analysis. (A) Correlation between IFIH1 and EIF2AK2 expression. (B) Correlation between EIF2AK2 and ISG15 expression. (C) Correlation between EIF2AK2 and STAT1 expression. (D) Correlation between IFIH1 and STAT1 expression. (E) Correlation between IRF7 and EIF2AK2 expression. (F) Correlation between IRF7 and IFIH1 expression. (G) Correlation between IRF7 and ISG15 expression. (H) Correlation between RSAD2 and IRF7 expression. (I) Correlation between IRF7 and STAT1 expression [file 40001_2025_2953_MOESM1_ESM.pdf]

Supplementary Material

Identification of differentially expressed immune-related genes in patients with Systemic Lupus Erythematosus and the development of a hub gene-based diagnostic model

Quangang Fang[1], Weili Kong[1], Huaping Zhou[1], Yilin Pang[2], Haiyun Liu[1*]

[1] Department of Laboratory, Jiangxi Provincial People's Hospital, The First Afliated Hospital of Nanchang Medical College, Nangchang 330000, China

[2] Zhejiang Provincial Key Laboratory of Medical Genetics, School of Laboratory Medicine and Life Sciences, Wenzhou Medical University, Wenzhou, Zhejiang 325035, China.

*Correspondence: Haiyun Liu, bingfengdepaomo@163.com

Supplementary Figures

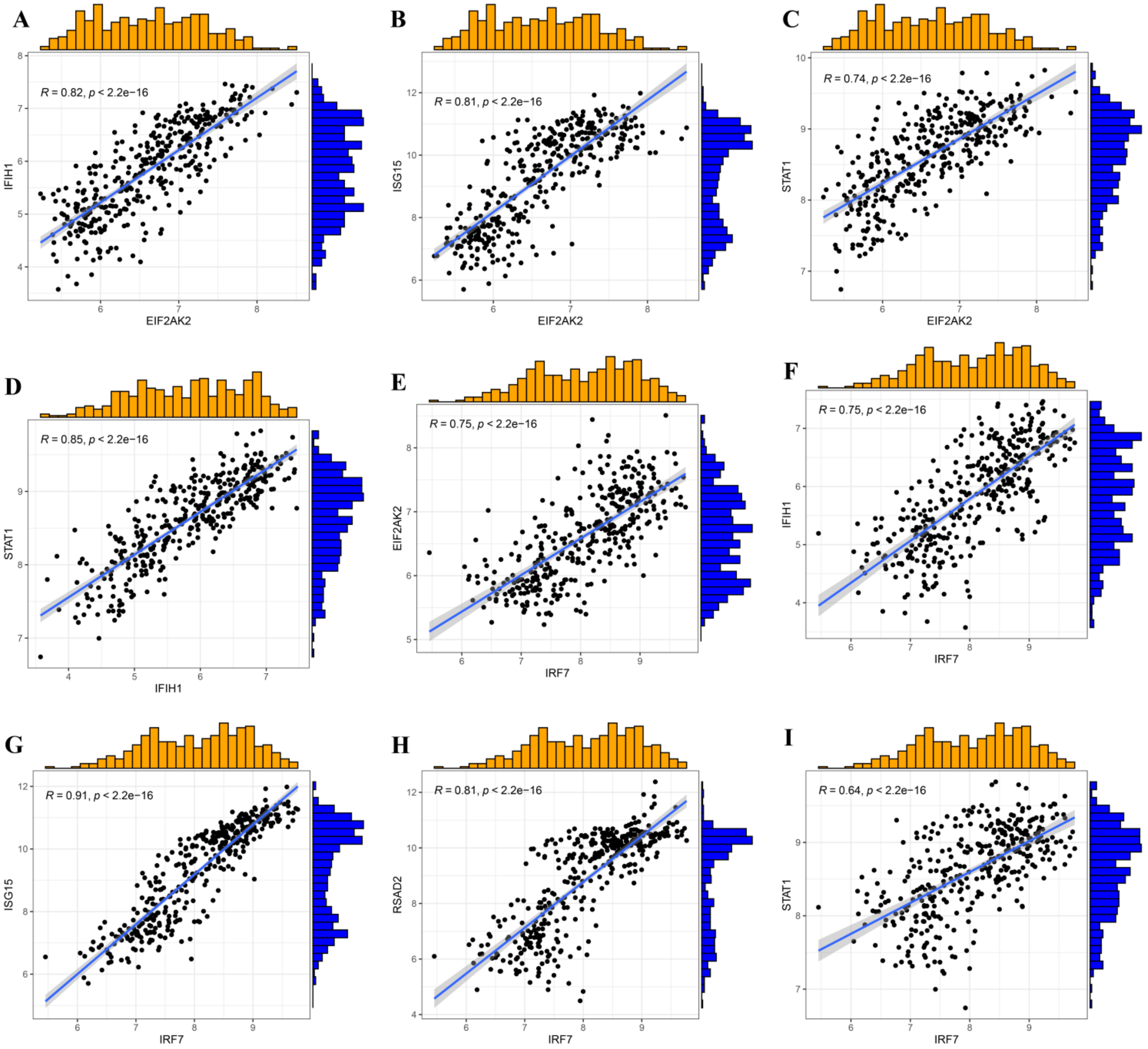

Supplementary Figure S1. Correlation analysis. (A) Correlation between IFIH1 and EIF2AK2 expression. (B) Correlation between EIF2AK2 and ISG15 expression. (C) Correlation between EIF2AK2 and STAT1 expression. (D) Correlation between IFIH1 and STAT1 expression. (E) Correlation between IRF7 and EIF2AK2 expression. (F) Correlation between IRF7 and IFIH1 expression. (G) Correlation between IRF7 and ISG15 expression. (H) Correlation between RSAD2 and IRF7 expression. (I) Correlation between IRF7 and STAT1 expression.

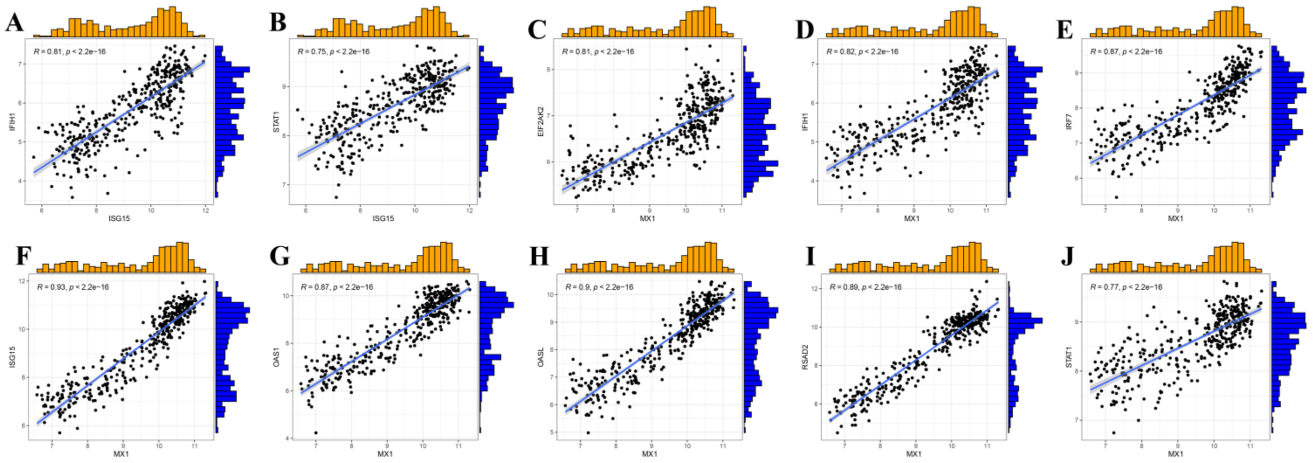

Supplementary Figure S2. Correlation analysis. (A) Correlation between ISG15 and IFIH1 expression. (B) Correlation between ISG15 and STAT1 expression. (C) Correlation between MX1 and EIF2AK2 expression. (D) Correlation between MX1 and IFIH1 expression. (E) Correlation between MX1 and IRF7 expression. (F) Correlation between MX1 and ISG15 expression. (G) Correlation between MX1 and OAS1 expression. (H) Correlation between MX1 and OASL expression. (I) Correlation between MX1 and RSAD2 expression. (J) Correlation between MX1 and STAT1 expression.

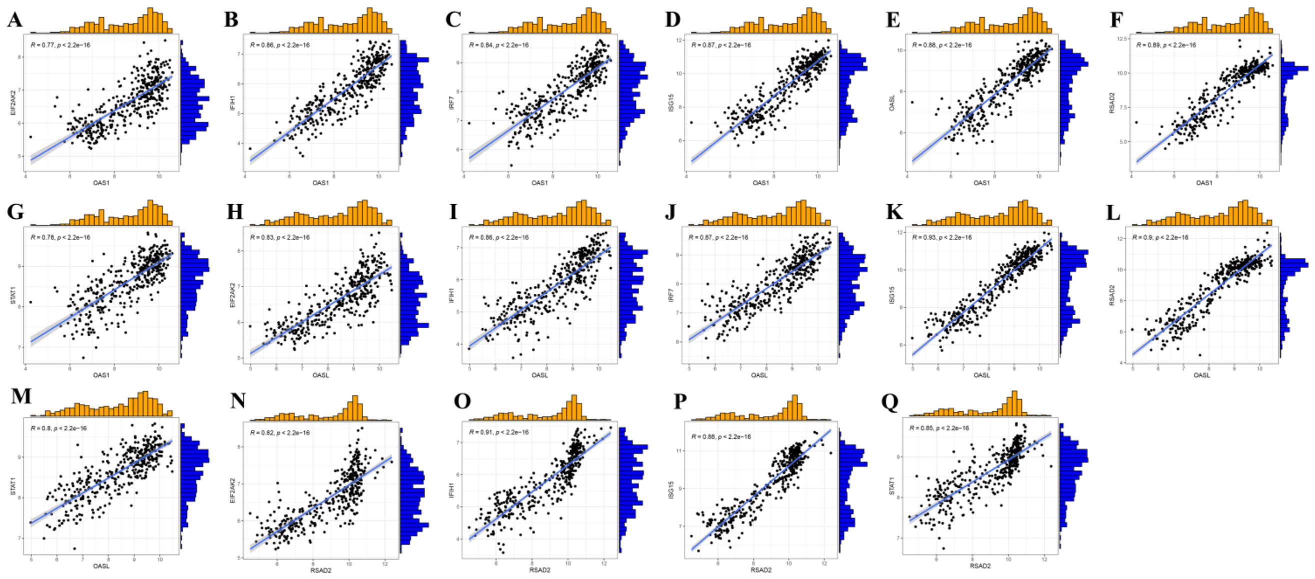

Supplementary Figure S3. Correlation analysis. (A) Correlation between OAS1 and EIF2AK2 expression. (B) Correlation between OAS1 and IFIH1 expression. (C) Correlation between OAS1 and IRF7 expression. (D) Correlation between OAS1 and ISG15 expression. (E) Correlation between OAS1 and OASL expression. (F) Correlation between OAS1 and RSAD2 expression. (G) Correlation between OAS1 and STAT1 expression. (H) Correlation between OASL and EIF2AK2 expression. (I) Correlation between OASL and IFIH1 expression. (J) Correlation between OASL and IRF7 expression. (K) Correlation between OASL and ISG15 expression. (L) Correlation between OASL and RSAD2 expression. (M) Correlation between OASL and STAT1 expression. (N) Correlation between RSAD2 and EIF2AK2 expression. (O) Correlation between RSAD2 and IFIH1 expression. (P) Correlation between RSAD2 and ISG15 expression. (Q) Correlation between RSAD2 and STAT1 expression.
